# Peptide based vesicles for cancer immunotherapy: design, construction and applications

**DOI:** 10.3389/fimmu.2025.1609162

**Published:** 2025-05-27

**Authors:** Yulin Yu, Jiaxin Lyu, Yizimujiang Muhadaisi, Chen Shi, Dongyuan Wang

**Affiliations:** ^1^ Department of Pharmacy, Union Hospital, Tongji Medical College, Huazhong University of Science and Technology, Wuhan, China; ^2^ Hubei Province Clinical Research Center for Precision Medicine for Critical Illness, Wuhan, China; ^3^ School of Pharmacy, Tongji Medical College, Huazhong University of Science and Technology, Wuhan, China

**Keywords:** cancer immunotherapy, peptide vaccine, vesicle, peptide based vesicle, anti-cancer peptide

## Abstract

Cancer immunotherapy has emerged as a powerful strategy for clinical treatment of malignant cancers. Despite the advances, cancer immunotherapy has met several challenges such as the limited efficacy to small subsets of patients, and serious autoimmune side effects. Cancer vaccines that target neoantigens to direct and amplify immune responses against tumors, have shown their efficacy and safety in preclinical and clinical researches. The developed cancer vaccines mainly contained peptide vaccines, mRNA vaccine, cell vaccine and oncolytic virus vaccine. In the last decade, both peptide based vaccines and vesicle based vaccines have attracted enormous attention for personalized vaccine development due to their potent efficacy in different tumor models. Peptide based vesicles are one kind of vesicles that are modified with functional peptides to enhance the efficiency of immune response and anti-cancer effect. In this review, we will introduce the basic characteristics, classification and biological application of vesicles or peptide based cancer vaccines respectively. Then the design and construction of peptide based vesicles will be summarized. Finally, we concluded the biological applications of peptide based vesicles in various cancer types and analyzed the key obstacles to overcome for their clinical applications. We hope this review could provide a better understanding of the construction of peptide based vesicles and their prospects for clinical applications.

## 1 Introduction

**Figure 1:**
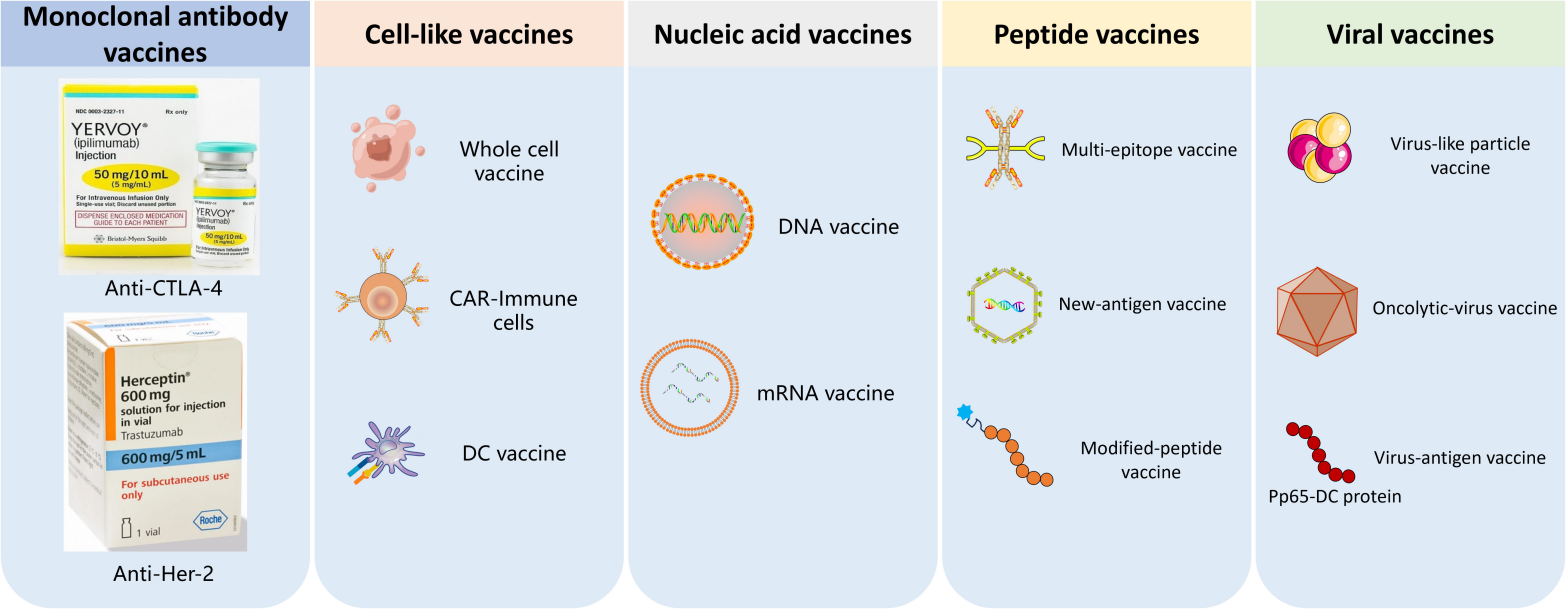
The classification of tumor vaccines. 1) Monoclonal antibodies, 2) Cell-like vaccines such as CRT-immune cells vaccines and DC vaccine; 3) Nucleic acid vaccines,; 4) Peptide vaccines; 5) oncolytic-virus vaccine and virus-antigen vaccine.

Immunotherapy aims to enhance the natural immune system to eliminate malignant cells. The advent of cancer immunotherapy has a profound impact on the field of cancer treatment, significantly prolonging the survival of patients with malignant tumors and improving their quality of life (1). However, few patients can benefit from the currently available immunotherapies and many patients suffer from serious immune-related adverse events (2). In recent years, various forms of immunotherapy showed great potential for cancer immunotherapy, such as CAR-T cell therapy, peptide vaccines, mRNA vaccines, dendritic cell (DC) vaccines, oncolytic viruses (OVs) et al., shown in **Figure 1** (3–5).

**Figure 2:**
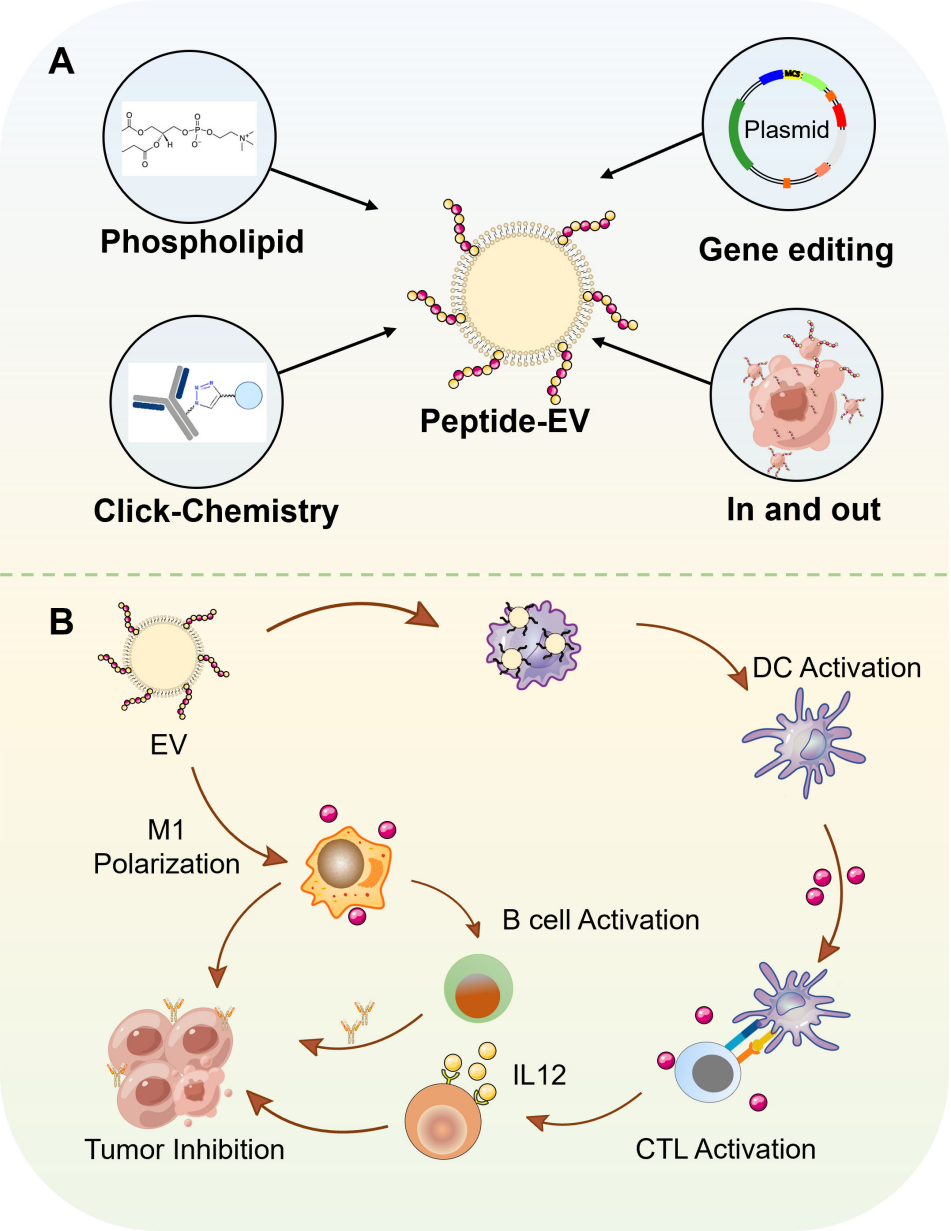
The various types of peptide-based vesicles and their application in cancer immunotherapy. **(A)** Preparation methods of peptide-based vesicles. 1) Phospholipid-modified peptide insertion. 2) Click chemistry-mediated conjugation. 3) Genetically engineered expression. 4) Endocytosis-exocytosis strategy. **(B)** Application of peptide-based vesicles in tumor immunotherapy. Peptide-based vesicles were internalized by DCs. Mature DCs present antigenic peptide-MHC complexes to naive T cells, activating cytotoxic T lymphocytes that migrate to tumor sites and induce apoptosis in malignant cells. In addition, M2-like tumor-associated macrophages uptake peptide-based vesicles, leading to their reprogramming into pro-inflammatory M1 phenotypes. M1 macrophages secrete cytokines to directly kill tumor cells. Furthermore, M1 macrophages further present tumor-associated antigens to B cells, promoting their differentiation into plasma cells.

Extracellular vesicles have been proven to contribute to the remodeling of the immune-suppressive tumor microenvironment (TME), thereby influencing the efficacy of immunotherapy. In order to satisfy different demands for cancer therapy, extracellular vesicles are engineered by various methods, among which peptide based vesicles exhibit significant potential, characterized by key benefits from functional peptides such as high specificity for tumor targeting, excellent biocompatibility, and robust immune regulatory capabilities (6). Thus, peptide based vesicles are modified by various peptides which can modify the vesicle surface to accurately recognize receptors on tumor cell membranes, thereby enhancing drug accumulation in tumor tissues (6). Meanwhile, peptide based vesicles possess the capacity to modulate Therapeutic Drug Monitoring (TDM). It can counteract the immunosuppressive state in TDM by regulating the functions of tumor-associated immune cells. For example, IL4RPep-1 peptide (CRKRLDRNC) modified exosomes can specifically target M2-type tumor-associated macrophages (TAMs) and facilitate their conversion into anti-tumor M1-type macrophages (7). Furthermore, peptide vesicles exhibit excellent compatibility with the human physiological environment and are less prone to induce immunological rejection. They also possess significant biodegradability. After completing their drug delivery mission, peptide vesicles can spontaneously degrade into harmless small molecules within the body, which are then eliminated through standard metabolic pathways. This prevents long-term accumulation and mitigates the risk of long-term toxicity associated with residual materials (7). The properties of peptide based vesicles make them as potential drug candidate for cancer therapy. In this review, we will first introduce the function of peptide vaccines and vesicle vaccines respectively, and then introduce the design and construction of peptide based vesicles, next review their anti-cancer application, and last discuss their challenges for clinical translation and potential solutions to overcome them, shown as **Figure 2**.

## 2 Roles of vesicles in cancer immunotherapy

### 2.1 Basic characteristics of extracellular vesicles

EVs are one kind of nanometer-sized spherical hollow structures that can carry bioactive molecules and deliver them to recipient cells (8). Classic EVs can be broadly classified into exosomes, microvesicles, and apoptotic bodies. Exosomes, with a diameter of 50–100 nm, can be released by resting or stimulated cells and can transfer mRNA, miRNA, and oncogenic receptors, exhibiting antigen presentation, immune activation, and immune suppression activities (9) (10). Microvesicles, measuring 100–1000 nm in diameter, are produced by platelets (11), red blood cells (12), or epithelial cells (13) through outward budding of cell membranes and have procoagulant functions. Apoptotic bodies, with a diameter of 1–5 micrometers, are generated during cell apoptosis and can transfer DNA and oncogenes, presenting T-cell epitopes to immune cells when ingested by phagocytes, thereby exerting immune suppressive effects (8).

### 2.2 Immunological functions of cellular vesicles

Although most cells possess the ability to produce extracellular vesicles (EVs), not all EVs derived from cells can be used as carriers for drug delivery. The standards for drug delivery included the production yield, surface protein properties, size, and the composition in the vesicles. Currently, several cell types have been explored as potential donor sources for EVs used in drug delivery, such as dendritic cells (DC) (14), macrophage (15), tumor cells (9), red blood cells et al. (16) (12).

Tumor cell-derived EVs (TEVs), especially autologous TEVs, carry a similar repertoire of tumor antigens, co-stimulatory molecules, and DNA fragments as their parental cells (17, 18). This property can elicit robust T-cell-dependent anti-tumor immune responses and has demonstrated therapeutic effects in melanoma mouse models (19), hepatocellular carcinoma (20), and colon cancer (21). Compared to EVs produced by non-cancerous cells, TEVs can achieve tumor cell-specific targeting through intrinsic homotypic adhesion mediated by membrane surface antigens (22). In tumor therapy, TEVs play a significant role, such as enabling deep tumor penetration for drug delivery (23), exhibiting high specific homing capabilities, and activating the signal transducer and activator of transcription 3 pathway (24). Additionally, the *in situ* generation of micron-sized tumor cell-derived vesicles serves as an autologous tumor vaccine to enhance systemic immune responses (25), and functional DNA-modified cancer cell membrane vesicles are used as targeted vaccines for tumor immunotherapy (26). However, the role of TEVs in promoting cancer progression by enhancing cell proliferation and evading apoptosis, inducing angiogenesis, metabolic reprogramming, enhancing invasion and metastasis, and evading immune surveillance has been well-documented (27). Therefore, unlike exosomes from other sources, TEVs can be a double-edged sword when used as therapeutic agents in cancer treatment. A thorough elucidation of their formation, secretion, and network functions is urgently needed to realize this attractive and promising cancer treatment strategy, requiring more extensive *in vivo* studies with larger sample sizes to investigate the effectiveness and safety of TEVs as future drug delivery systems (DDS) (28).

Dendritic cells (DCs) are fundamental immune cells essential for antigen presentation and T cell activation. DC-derived vesicles (DEVs) maintain the basic immune stimulating ability of DCs (e.g., antigen presentation to T cells) (29). DEVs production processes are amenable to strict regulation and monitoring (e.g., easy determination of their composition and MHC-I and MHC-II contents) and pose lower risks associated with feasible cell or viral therapies (e.g., *in vivo* replication risks) (30, 31). In recent decades, DEV-based therapies have been widely applied in immunotherapy and drug delivery. For instance, in breast cancer treatment, DEVs can enhance cancer cell sensitivity to immune checkpoint inhibitors and prevent recurrence of resected tumors (15). DEVs can overcome biological barriers like the blood-brain barrier (BBB), making them attractive for future drug delivery (32).

Macrophage-derived EVs express functional immune regulatory proteins including MHC class I and II (33), preferentially inducing Th1-type (cell-mediated) immune responses that direct T cells to attack abnormal cells (e.g. cancer cells) or cells infected by intracellular parasites (34, 35). Macrophage-derived EVs also have extensive applications in tumor treatment. For example, macrophage-derived exosomes are thought to transfer miR-365, a key regulator of gemcitabine resistance in pancreatic cancer (36). M1-like macrophage-derived EVs (M1 EVs) are used to treat glioblastoma multiforme (37). In photodynamic therapy (PDT), fusion of M1 EVs with thylakoid membranes of natural plants imparts active tumor targeting ability to M1 EVs. Therefore, macrophage-derived EVs offer promising new strategies for tumor treatment.

Besides these cells, there are other candidates for drug delivery vesicles, such as those derived from red blood cells (RBCs) (38, 39), natural killer (NK) cells (40) and T cells (41). The CD47 on RBC-derived EVs interacts with its receptor, signal regulatory protein alpha (SIRPa) on macrophages, protecting the RBC-derived EVs from clearance by initiating a “don’t eat me” signal (42). NK cell-derived EVs contain tumor necrosis factor-α and granzyme B, exhibiting cytotoxic effects on glioma cells (40) and melanoma- cells (43) with no significant side effects *in vitro* and *in vivo*. Furthermore, studies have found that activated CD8^+^ T cells from healthy mice release cytotoxic EVs, leading to a significant reduction in tumor invasion and metastasis (44). EVs derived from CD4^+^ T cells enhance the anti-tumor response of CD8^+^ T cells by augmenting their proliferation and activity without affecting regulatory T cells.

Among various sources of EVs, RBCs exhibit distinct advantages in safety and scalable production due to their relatively low content of cellular components and ease of procurement (45). TEVs, while amenable to scalable production through *in vitro* expansion of tumor cells, possess surface markers enriched with tumor-specific antigens that may enhance tumor-targeting efficacy (23). However, their content (proteins, nucleic acids, etc.) may carry oncogenic risks, necessitating further improvements in biosafety (46). Immune cell-derived EVs (e.g., DCs, macrophages, or NK cells) retain functional biological properties inherent to their parent cells, enabling tailored therapeutic applications. Nevertheless, the scalability of immune cell-derived EVs remains constrained due to stringent ex vivo expansion requirements and high costs associated with isolating immune cells from biological systems (47). These factors collectively highlight the need to balance source-specific advantages with technical and safety considerations for clinical translation.

## 3 The role of peptides in tumor immunotherapy

### 3.1 Peptides as antigens

Peptide vaccines can be divided into two categories based on their activation functions, one group stimulates the innate immune system by interacting with tumor-associated macrophages (TAM), dendritic cells (DC), neutrophils, and natural killer (NK) cells, while the other group can activate the adaptive immune system by interacting with T cells and B cells (48–50).

For tumor-associated macrophages (TAM) in the innate immune system, TAMs exhibit two phenotypic activation states: the antitumor M1 and the protumor M2 (51–55). Currently, the main strategies to block M2-TAM activity involve inhibiting the recruitment of macrophages to tumors and converting M2-TAMs to M1-TAM. For example, researchers developed a biohybrid material with the ability to immunologically regulate TAM cell populations, using vascular endothelial growth factor (VEGF) mRNA interference-M2 targeting peptide. This material primarily blocks M2-TAM activity and cancer cell growth by inhibiting VEGF-related signaling pathways and triggering host immune responses that lead to sustained tumor regression, and it can also generate long-lasting antitumor immune memory (56).

As for DCs, Wang et al. selected the TRP2 peptide and the dodecamer CPP (AAVLLPVLLAAP) to prolong the presentation of MHC class I-restricted self-peptides on dendritic cells (DCs), thereby enhancing antitumor immune responses. CPP1 can effectively deliver the TRP2 peptide into mature DCs and retain the full capacity of DCs to present MHC-peptide complexes to antigen-specific T cells over an extended period. They demonstrated that immunizing mice with DCs loaded with TRP2-CPP1 conjugate led to complete protection against B16 tumor suppression, and lung metastasis inhibition (57).

The human body has three principal subtypes of mature T cells: cytotoxic T lymphocytes (CTLs), helper T cells, and regulatory T cells (Tregs).Immune checkpoint blockade is one of the major immunotherapies (58–64), which precisely targets tumor cells by blocking immune checkpoints such as CTLA-4 and PD-1. Researchers have discovered peptides that inhibit the PD-1/PD-L1 interaction and reactivate T cell function against tumor cells, including peptide-57, CLP001/CLP002, and PD-L1 Pep-1/PD-L1 Pep-2. These peptides not only reawaken T cells via their PD-L1 inhibiting activity but also utilize PD-L1 as a tumor target to deliver chemotherapeutic agents specifically to tumors exhibiting elevated PD-L1 expression.

### 3.2 Peptides as immune modulators

Anti-cancer peptide(ACP) are bioactive peptides that inhibit cancer cell growth via a spectrum of mechanisms. Lytic peptides are toxic molecules that kill cancer cells by disrupting cell membrane. Recent studies revealed that the cell fragments by lytic peptides can act as tumor antigens to trigger the immune response. For example, lytic peptide EP-100 offers a unique therapeutic option for patients demonstrating insufficient responses to immunotherapy for ovarian cancer (65). EP-100 is a synthetic fusion peptide composed of an LHRH ligand and a lytic peptide (CLIP-71) that specifically binds the LHRH receptor (LHRH-R) (66). As an immune enhancer, it induces PD-L1 synthesis in neoplastic cells, therefore altering the tumor microenvironment. This leads to an augmentation of immune cells that facilitate tumor lysis (CD8^+^ T cells, NK cells, dendritic cells, and macrophages) while diminishing immunosuppressive cells (Tregs, B cells, and mMDSCs). Targeted ACPs can inhibit immune-related signal pathways to modulate immune response. For example, A new peptide-based PROTAC has been developed to combat the prevalent resistance to PD-1/PD-L1 inhibitors in clinical contexts by degrading PD-1 or PD-L1, thus inducing cancer cell apoptosis (67).

### 3.3 Application of peptides as carriers in tumor immunotherapy

Peptide nanoparticles are widely acknowledged as an effective approach in cancer immunotherapy because of their exceptional stability and significant capability for delivering peptide antigens and immunological adjuvants (68, 69). Peptides and their derivatives can self-assemble into one-dimensional fibers or nanofibers, which can then interlace to form hydrogels or nanoparticles, enabling the targeted release of peptides and adjuvants (69). Collier et al. developed a vaccine utilizing the Q11 self-assembling domain (ac-qqkfqfqqfeqq-am) produced from chicken ovalbumin (OVA323-339) to incorporate MUC1-derived peptides (70). These immunizations stimulate the production of potent antibodies specifically targeting breast cancer cells. To improve the application of this technique in clinical therapy, Huang et al. created a novel synthetic self-adjuvant vaccine using a self-assembling Q11 domain (71). This vaccine can produce fibrous structures under mild conditions and display multivalent B-cell epitopes, thereby markedly enhancing their immunogenicity.

## 4 The role of peptide-based vesicle for cancer immunotherapy

Vesicles play dual roles in immune activation and anti-tumor treatment by serving as an autologous tumor vaccine to enhance systemic immune responses and a good vehicle for drug delivery. However, the limited tumor selectivity and immune stimulating ability have hindered their broad applications. As we mentioned above, functional peptides can act as warheads for tumor selective penetration, as peptide vaccines to enhance tumor immune response, as immune modulators to inhibit immune-related signal pathways. These properties can be used to overcome the limitations of vesicle based application. In this part, we will introduce the construction and applications of peptide based vesicles, particularly the roles of peptides in vesicles to enhance therapeutic effect.

### 4.1 Forms of peptide-based vesicle

The principal techniques for constructing peptide-based vesicles encompass direct loading via phosphatidylation or click chemistry; surface modification of gene-edited cellular vesicles; and the administration of peptides to immune cells, followed by the preparation of vesicles from these cells to commence the antigen presentation process (72).

Zhu et al. chemically crosslinked a dibenzobicyclooctyne (DBCO) moiety to the surface of dendritic cell-derived extracellular vesicles (EVs) and subsequently reacted it with azide-functionalized MUC1 glycopeptide by click chemistry, thereby covalently affixing MUC1 to the EV surface (73). As for lipid insertion, Ye et al. introduced a noteworthy methodology (74). The 4F-KLA-LDL peptide was synthesized by combining the pro-apoptotic peptide KLA with an LDL-targeting peptide, which selectively binds to the overexpressed LDL receptors on blood-brain barrier (BBB) and glioblastoma (GBM) cell lines. Based on the molecular recognition between phospholipids on EV and ApoA-I mimetic peptides, They developed methotrexate (MTX)-loaded EVs functionalized with 4F-KLA-LDL peptide, which can target low-density lipoprotein (LDL) on GBM cells and enhance the transport of the pro-apoptotic peptide KLA and methotrexate (MTX) to U87 glioma cells. As for genetic manipulation, EVs are typically equipped with these peptides in EV donor cells using transfection or retroviral/lentiviral infection (75). For instance, Ohno et al. reported a technique involving the expression of a fusion protein within HEK-293T cells using a retroviral plasmid (76). This fusion protein consists of the transmembrane domain of the platelet-derived growth factor receptor and a peptide that targets the epidermal growth factor receptor (EGFR), resulting in EGFR-targeted extracellular vesicles (EVs). These electric vehicles are engineered to transport the anti-cancer miRNA let-7 straight to breast tumor cells. As for the chemical engineering approach to covalently conjugate peptides to EVs, Nakase et al. chemically synthesize stearyl-modified octaarginine peptide solid-phase peptide synthesis. The stearyl group functioned as an anchoring unit for membrane insertion. This method facilitated straightforward alteration of the exosome membrane to promote macropinocytosis, markedly increasing cellular absorption of extracellular vesicles (EVs) and enabling efficient intracellular transport of the artificially encapsulated ribosome-inactivating protein saporin through EVs, therefore resulting in tumor cell apoptosis (77). Nevertheless, the severe chemical treatment of EV surfaces, which may result in detrimental functional degradation, has hindered the widespread adoption of these methods (78). As for affinity conjugation, there are various methods that use EV-binding peptides or antibodies to coat EVs. However, these conjugations are transient and unstable. He et al. designed chiral peptide Au (I) infinite covalent polymers (DPAICP) using D-peptides and Au³^+26^. They then ultracentrifuged milk-derived extracellular vesicles (ME) membranes with lactoprotein, embedded the chiral peptide supramolecular assemblies into the ME membrane, and obtained an artificial milk DPAICP@ME with pharmaceutical and absorbable properties. This approach restores the p53 signaling pathway for cancer therapy while further activating T cells and enhancing the efficacy of anti-PD-1 immunotherapy (79). As mentioned earlier, existing surface modification methods for EVs have various drawbacks in terms of safety, stability, and integrity (80). A stable and gentle method for EV coupling has been developed by Pham et al. who devised a novel technique utilizing protein ligases (including sortase A and OaAEP1 ligase) to covalently attach EVs to high-copy-number targeting moieties. The conjugation of EVs with EGFR-targeting peptides or anti-EGFR nanobodies facilitates their accumulation in EGFR-positive cancer cells both *in vitro* and *in vivo*. Furthermore, this methodology is applicable for conjugating EVs with peptides and nanobodies targeting other receptors, such as HER2 and SIRPα (81).

### 4.2 Anti-cancer applications of peptide-based vesicle

Different peptide based vesicles had quite different functions for cancer immunotherapy, which are concluded in **Table 1**. The synergistic effect of peptide based vesicles can be categorized into three parts: enhancing immune response by peptide antigen or targeted peptide, augmenting anti-cancer effect of toxic peptides, improving tumor targeted delivery by peptide ligands.

**Table 1:** Summary of peptide based vesicles.

Peptide based vesicles	Peptide function	Vesicle resources	Immunological effects
E7p-OMVs	HPV-specific targeting peptide	E. coli	Effectively delivers peptide antigens to APCs, stimulates DC maturation, and induces peptide antigen-specific CD4^+^ Th1 and CD8^+^ CTL responses, thereby inhibiting the development of HPV-associated tumors and increasing the number of CD80^+^ and CD86^+^ DC cells.
T140p-KLAp-EV	T140 peptide and KLA peptide	RBC EVs	Enhances the specific apoptotic effects of KLA peptides in CXCR4-positive leukemia cells.
DPAICP@ME	Chiral peptide Au(I) involving organic thiols and Au³^+^	Milk-derived extracellular vesicles (ME)	Restores the p53 signaling pathway and further activates T cells, thereby enhancing the efficacy of anti-PD-1 immunotherapy.
IL4Rp1-DCEVs	IL4RPep-1 peptide (CRKRLDRNC)	Dendritic cell EVs	Reprograms IL4r-high and M2-polarized TAMs into an M1-like phenotype, thereby inhibiting tumor progression.
4F-KLA-LDLp-EVs	ApoA-I mimic peptide (4F-KLA-LDL peptide)	Extracellular vesicles (EVs) encapsulating the anticancer drug MTX	Improves receptor-mediated internalization and optimizes the transport of the pro-apoptotic peptide KLA and methotrexate (MTX) to U87 glioma cells.
Angp-TATP-SEVs	Ang peptide and TAT peptide	Small extracellular vesicles (sEVs)	Mainly involved in drug delivery across the blood-brain barrier and glioma, as well as strong tumor penetration effects.
OMVMPI-N, OMVMPI-SP,OMVMPI-C	MPI fusion peptide, OmpA signal peptide SP	OMVs	Used for immunomodulatory chemotherapy in bladder cancer.

#### 4.2.1 Enhancing immune response

The immune response of vesicles can be enhanced by peptide antigens or PD-L1 targeting peptides. For example, in cervical cancer, E7p-OMVs, entails the introduction of a plasmid encoding the peptide antigen E7p (amino acids 44-62) with CTL and Th cell epitopes into E. coli cells (6). This method facilitates the *in vivo* creation of E7p-encapsulated natural bacterial outer membrane vesicles (OMVs), which effectively transport peptide antigens to antigen-presenting cells (APCs), therefore impeding the progression of HPV-associated malignancies (6). In osteosarcoma, Wu et al. discovered that the interaction between NPM PD-L1 and IGFBP3 activates mTOR signaling and promotes osteosarcoma tumor growth through PGK1-mediated phosphorylation enhancement (82). They generated a PD-L1 phosphorylation-mimetic peptide incorporating the S279 location and encapsulated it within cRGD-modified RBCM vesicles to create peptide@cRGD-M. An effective peptide@cRGD-M nanoparticle method for osteosarcoma treatment was created by integrating erythrocyte membrane therapy with peptide therapy, thereby enhancing the anti-cancer effect.

#### 4.2.2 Augmenting anti-cancer effect of toxic peptides

In bladder cancer, Ren et al. reported a bioengineered OMV-based platform using bacterial OMVs as nanocarriers to encapsulate toxic MPI fusion peptides generated by genetic engineering (83). MPI was conjugated to both the C- and N-termini of the fusion peptide to facilitate membrane integration. As MPI-N could not be encapsulated by OMVs, EVs were utilized to encapsulate MPI-N, which was introduced with the OmpA signal peptide SP. Three bioengineered outer membrane vesicles (OMVs) were ultimately produced: OMVMPI-N (with minimal MPI-N), OMVMPI-SP (with MPI-N obstructed by SP), and OMVMPI-C. These were utilized for immunomodulatory chemotherapy in bladder cancer, resulting in good therapeutic outcomes and biosafety. In leukemia, T140-KLA-EV was synthesized by covalently attaching T140 and KLA peptides to pre-existing RBCEV membrane proteins utilizing OaAEP1Cys247Ala. This construct diminishes the infiltration of leukemia cells in the spleen by augmenting the specific apoptotic effects of KLA peptides in CXCR4-positive leukemia cells, consequently decelerating disease progression and improving overall survival (84).

#### 4.2.3 Improving tumor targeted delivery by peptide ligands

In glioblastoma, it has been documented that neuron-specific rabies virus glycoprotein (RVG) peptide-modified sEVs provide an efficient tissue-targeting delivery mechanism for the treatment of glioblastoma and Alzheimer’s disease (85, 86). Additionally, Zhu et al. developed functional Ang/TAT-sEVs-Dox by modifying sEVs with Ang peptide and TAT peptide (87). This system targets the blood-brain barrier and glioblastoma, penetrating both the barrier and the tumor. In lung cancer, Pham et al. conjugated RBCEVs with EGFR-targeting peptides using sortase A and OaAEP1 ligase (81). This method facilitates the targeted absorption of RBCEVs by EGFR-positive cells. Additionally, RBCEVs treated with paclitaxel (PTX) demonstrated substantial antitumor efficacy at low dosages (10–20 times lower than therapeutically equivalent doses) against EGFR-positive lung cancer. This technique is likewise pertinent to the conjugation of extracellular vesicles with peptides and nanobodies that target alternative receptors (e.g., HER2 and SIRPα) for precise medication delivery to pertinent malignancies. In prostate cancer, Diao et al. reported a novel strategy using cationic membrane-penetrating peptide TAT to encapsulate siRNA into EVs (87). Three TAT peptides were co-expressed with DRBD as a 3TD (TAT-TAT-TAT-DRBD) chimeric protein. The sequence-independent binding of DRBD enabled the multiplexing of siRNA for targeting several genes, yielding more potent therapeutic effects compared to single-gene targeting inhibitors. The concurrent siRNA-mediated silencing of the FLOH1, NKX3, and DHRS7 genes demonstrated considerable promise for enhancing CRPC treatment, offering a novel approach for CRPC therapy.

## 5 The challenges and future direction of peptide based vesicles

Peptide based vesicles, as an innovative approach for cancer immunotherapy, have shown considerable promise in drug delivery and immunotherapeutic applications. Nonetheless, their clinical application encounters several obstacles, including safety, immunogenicity, stability, targeting ability, drug releasing, size and product preparation and manufacturing. Safety stands as the paramount concern in advancing peptide based vesicles toward clinical applications. Cell-derived vesicles inherently carry biological information from their parent cells, which endows these vesicles with unique biological functions while simultaneously introducing potential safety hazards. For instance, vesicles originating from tumor cells carry genetic material from their parent tumor cells, posing a latent carcinogenic risk. Current research strategies predominantly focus on isolating exosomes or fabricating vesicles through cell membrane extraction. However, these methodologies inevitably amplify procedural complexity and compromise product uniformity, thereby representing significant challenges in therapeutic development. As for the intrinsic low immunogenicity of peptide vesicles, combination therapy could be a method to overcome it. Currently, researchers have identified many peptides with strong affinity for PD-L1 or CTLA-4 using phage display method. These peptides can be affixed to the surface of vesicles and transported to the tumor microenvironment. Peptide vesicles can transport immunomodulatory molecules, such as cytokines or short interfering RNAs, and deliver them to the tumor microenvironment via targeted administration, thereby altering the immune milieu. Concerning the scalability of peptide-vesicle conjugates, enzymatic techniques exhibit a certain degree of transformability for extracellular vesicles produced from alternative cellular sources (81). This presents novel concepts for the synthesis of various peptide-vesicle conjugates and offers direction for the formulation of peptide-vesicle combinations. In addition, size and product preparation and manufacturing is an essential factor to consider, the current vesicle separation method is mainly ultracentrifugation, however, this method is expensive and the sample processing capacity per batch is limited. The dimensions of peptide based vesicles substantially influence their *in vivo* dispersion and targeting efficacy. Larger vesicles may encounter difficulties in traversing the thick tumor extracellular matrix (ECM), whereas smaller vesicles may be swiftly eliminated. Furthermore, size heterogeneity may result in unpredictable medication release. To tackle these challenges, various strategies may be employed: optimizing synthesis processes, such as solvent-switching or self-assembly techniques, to accurately regulate vesicle size, creating intelligent responsive designs that leverage pH or temperature variations to modulate vesicle behavior, or utilizing nanoencapsulation to improve stability and control release.

## 6 Conclusion and discussion

Peptide-based vesicles have versatile roles in cancer immunotherapy due to the incorporation of peptides into vesicles, including the immune checkpoint blockade, modulating the tumor microenvironment, enhancing their delivery specificity, activating immune cells et al. As extracellular vesicles (EVs) lack target-specificity, peptide ligands targeting cancer cell surface can be used for efficient EV delivery. For example, Tin et al. conjugated EVs with an epidermal growth factor receptor (EGFR)-targeting peptide and found EGFR targeting EVs facilitates their accumulation in EGFR-positive cancer cells both *in vitro* and *in vivo*. This peptide based vesicles significantly increases drug efficacy in a xenografted mouse model of EGFR-positive lung cancer at a low dose (81). The anti-cancer peptides can also be loaded into vesicles to enhance their cancer immunotherapeutic effects. For example, Tang et al. developed cRGD-functionalized chimaeric vehicle for LTX-315 delivery, which in combination with CpG adjuvant and anti-PD-1 boost immunotherapy of malignant B16F10 melanoma in mice (88). This combination was proved to secret IL-6, IFN-γ and TNF-α, tumor infiltration of CD8^+^CTLs and Th, and induction of TEM and TCMin spleen. Peptide antigens are good tools to enhance the cancer immunity of vesicles. For example, peptide antigen E7p modified EVs could effectively transport peptide antigens to antigen-presenting cells (APCs), promote dendritic cell (DC) maturation, and elicit peptide antigen-specific CD4^+^ T helper 1 (Th1) and CD8^+^ cytotoxic T lymphocyte (CTL) responses, therefore impeding the progression of HPV-associated malignancies.

This article provides a detailed overview of the applications and underlying mechanisms of vesicles from different cell sources in cancer therapy, as well as the application of peptides with immune activation and modulation functions in cancer treatment. From preparation methods to application mechanisms, the research on peptide-vesicle composite carriers in cancer immunotherapy is further explored. However, despite extensive research by many scholars on the application of peptide-vesicle composite carriers in cancer treatment, their clinical application still faces many obstacles. Future research should focus on how to further improve targeting to tumor tissues, enhance biocompatibility, simplify the formulation process, streamline storage and transportation conditions, and improve biosafety. These challenges need to be overcome through further research and technological innovation to promote the successful clinical application of peptide-modified vesicles.
